# Building a Comprehensive Mill-Level Database for the Industrial Sectors Integrated Solutions (ISIS) Model of the U.S. Pulp and Paper Sector

**DOI:** 10.1371/journal.pone.0120954

**Published:** 2015-03-25

**Authors:** Nabanita Modak, Kelley Spence, Saloni Sood, Jacky Ann Rosati

**Affiliations:** 1 Oak Ridge Institute for Science and Education (ORISE) Postdoctoral Fellow, Office of Research and Development, Air Pollution Prevention and Control Division, U.S. Environmental Protection Agency, Research Triangle Park, North Carolina, United States of America; 2 Office of Air Quality Planning and Standards, U.S. Environmental Protection Agency, Research Triangle Park, North Carolina, United States of America; 3 Student Contractor, Office of Research and Development, U.S. Environmental Protection Agency, Research Triangle Park, North Carolina, United States of America; 4 Office of Research and Development, U.S. Environmental Protection Agency, Research Triangle Park, North Carolina, United States of America; US Army Engineer Research and Development Center, UNITED STATES

## Abstract

Air emissions from the U.S. pulp and paper sector have been federally regulated since 1978; however, regulations are periodically reviewed and revised to improve efficiency and effectiveness of existing emission standards. The Industrial Sectors Integrated Solutions (ISIS) model for the pulp and paper sector is currently under development at the U.S. Environmental Protection Agency (EPA), and can be utilized to facilitate multi-pollutant, sector-based analyses that are performed in conjunction with regulatory development. The model utilizes a multi-sector, multi-product dynamic linear modeling framework that evaluates the economic impact of emission reduction strategies for multiple air pollutants. The ISIS model considers facility-level economic, environmental, and technical parameters, as well as sector-level market data, to estimate the impacts of environmental regulations on the pulp and paper industry. Specifically, the model can be used to estimate U.S. and global market impacts of new or more stringent air regulations, such as impacts on product price, exports and imports, market demands, capital investment, and mill closures. One major challenge to developing a representative model is the need for an extensive amount of data. This article discusses the collection and processing of data for use in the model, as well as the methods used for building the ISIS pulp and paper database that facilitates the required analyses to support the air quality management of the pulp and paper sector.

## Introduction

In response to a 2004 recommendation by the National Research Council [1], the U.S. Environmental Protection Agency (EPA) is developing integrated assessment techniques to monitor and manage environmental protection. These techniques include multi-pollutant, sector-based approaches to assessing and regulating air quality. A mathematical modeling framework can be used to facilitate these analyses and, accordingly, the Industrial Sectors Integrated Solutions (ISIS) model is under development. The ISIS model is a multi-sector, multi-product dynamic linear modeling framework that evaluates the economic impact of emission reduction strategies for multiple air pollutants. The model focuses on air pollutants, and does not assess wastewater or solid waste disposal. The model analyzes and evaluates the overall environmental and economic performance for a particular industrial sector. A case study using the ISIS-cement model can be found on the EPA’s Technology Transfer Network [2] or in chapter eight of “Global Climate Change—The Technology Challenge” [3].

The ISIS model considers many facility-level economic, environmental, and technical parameters, as well as sector-level market data. Facility-level economic data such as fuel costs, capital costs, production costs (e.g., labor, raw materials, maintenance), and add-on control costs are needed to evaluate the financial impacts of process changes and add-on control devices associated with the implementation and enforcement of environmental regulations. Facility-level environmental data such as types of emission units, categories and quantities of pollutants emitted, types of add-on controls and process modifications used to reduce emissions, and the amount of emission reduction achieved with each control or modification, are needed to evaluate the current environmental impacts of the facilities as well as potential emission reductions strategies associated with implementing air regulations. Other technical facility-level data such as production capacities, process types, fuel utilization, and product compositions and their corresponding energy intensities are also needed to characterize each facility. Finally, sector-level market data such as product market prices, import demand, and export demand are needed to assess the impact of regulations on the U.S. and global market. This article describes the method used to construct the ISIS database for the pulp and paper industrial sector, including the type of data needed, data processing, data analysis, and relevant assumptions that were made to develop the database to be used as the input to the ISIS model. All references to the unit “ton” in this paper refers to short ton unless otherwise specified.

### The Pulp and Paper Industrial Sector

The U.S. pulp and paper industry is a diverse sector that utilizes a variety of processes, and manufactures hundreds of different grades of paper [4]. The industry is grouped under paper manufacturing in the North American Industry Classification System (NAICS) as code 322 [5] and includes pulp, paper, and paperboard mills (NAICS code 3221) and converted paper product manufacturing (NAICS code 3222). Facilities in the 3221 NAICS code classification are primarily engaged in producing pulp and/or paper and paperboard. A facility producing both pulp and paper, with paper as the primary product, is considered an integrated facility. Processes and emissions from these different types of facilities vary greatly, and in order to accurately represent the industrial sector, the model will need to account for them.

Integrated facilities generally produce paper in six main processing steps: wood preparation, pulping, pulp washing, pulp screening, bleaching (optional, depends on final product type) and paper making [6]. Facilities utilizing the chemical pulping process use chemical recovery to recover pulping chemicals and generate process steam. The wood preparation process involves wood cutting, transporting, debarking, chipping, and screening of the raw material. The main objective of this process is to produce uniformly sized chips to be used in the pulping process, a process in which wood is broken down into fibers that can be used for papermaking. There are three main types of pulping: mechanical, chemical, and semichemical. In the case of chemical pulping, cellulosic fibers are separated from the cellulose-hemicellulose-lignin matrix in wood using high temperature, pressure, and chemicals. In the case of mechanical pulping, logs or chips are mechanically broken into smaller pieces. Semichemical pulping uses a combination of both methods. From the pulping process, pulp and spent cooking liquor proceed to pulp washing. The pulp washing process is used to remove cooking chemicals and the dissolved wood components in the cooking liquor for recovery and for energy generation, respectively. The recovery of these materials also minimizes the addition of chemicals and solids to the effluent treatment plant. The pulp screening process separates cooked pulp fibers from uncooked fiber bundles and knots. The pulp proceeds from screening to bleaching if the final product requires bleached pulp, or to the papermaking process if the final product utilizes unbleached pulp. The bleaching process involves removing the lignin that still remains after cooking (chemically whitening) or breaking double bonds in the lignin without removing it (brightening), as the lignin contains the chromophoric groups which make the pulp dark. Bleached pulp proceeds to the papermaking process.

The papermaking process involves stock preparation, dewatering, pressing, drying and finishing [6]. First, pulp fibers are treated mechanically by refining to produce flexible fibers suitable for papermaking. The treated fibers are blended with product specific additives (e.g., fillers for printing grades, or wet strength agents for tissue) and are diluted significantly with water to create the papermaking slurry. This slurry is processed on a paper machine, which creates a fiber mat and removes water by gravity, suction, pressure, and heat. The rolls of paper produced on the paper machine can be converted to final products on site, or may be shipped to another location for conversion.

The chemical recovery process involves evaporation, combustion, causticizing, and calcining [6]. The weak black liquor from pulp washing is processed using a multiple-effect evaporator system to increase the black liquor solids content by removing water. This is done to improve the heating value of the liquor, because it will be burned in a recovery furnace to generate steam. The purpose of the recovery furnace is to burn the organics in the black liquor and recover the inorganics in molten form. These inorganics (known as smelt) are dissolved to create green liquor. Green liquor is then clarified and causticized using lime to create white liquor for the pulping process. Lime mud is collected from the white liquor clarifier and burned in a lime kiln to regenerate lime for the caustization process. All of the process steps discussed in this section are potential air emission points that are considered in regulatory review and development.

### Current Economic Status of the Sector

**Fig 1:**
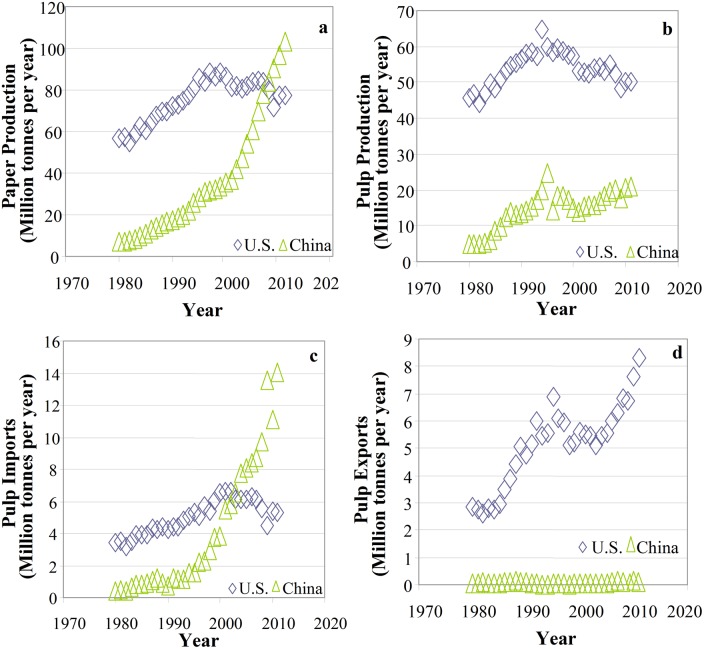
Historical pulp and paper trends for the U.S. and China.

Pulp and paper production has been prevalent in the U.S. since the early 1900s, and continues to contribute significantly to the U.S. economy. Market research indicates that the pulp, paper, and paperboard industry produced $80 billion in revenue in the U.S. in 2011 [7]. Historical production data were obtained from the Food and Agriculture Organization of the United Nations FAOSTAT database for “pulp for paper” and “paper and paperboard” [8]. In 2011, the most recent year with data available, the five highest world producers of pulp for paper were the U.S. (50.2M tonnes), China (21.1M), Canada (18.3M), Brazil (13.9M), and Sweden (11.7M). In 2011, the five highest world producers of paper and paperboard were China (103.1M tonnes), the U.S. (77.4M), Japan (26.2M), Germany (22.7M), and Canada (12.1M). Historically, the U.S. and China have led pulp and paper production. China production of paper began to significantly increase in 2002 and surpassed U.S. production in 2008, as shown in Fig. 1a. Pulp production did not follow this trend, as the U.S. maintained a significantly higher pulp production for all of the years analyzed (1980–2011), as shown in Fig. 1b. As expected with China’s large increase in paper production but minimal increase in pulp production, China’s pulp imports significantly rose from 2002 to 2011 (Fig. 1c). The U.S.’s exports of pulp also increased during this time (Fig. 1d) [8]. This interaction is important for understanding environmental impacts of pulp production.

### Environmental Regulations

Air emissions from the U.S. pulp and paper industry have been federally regulated since the 1978 Kraft Pulp Mill New Source Performance Standards (NSPS) [9]. This regulation controlled the total reduced sulfur (TRS) emissions and particulate matter (PM) emissions from pulp mill and chemical recovery emission units. The National Emission Standards for Hazardous Air Pollutants (NESHAP) for Pulping and Papermaking [10] was promulgated in 1997 and controlled hazardous air pollutant (HAP) emissions from pulping and papermaking sources. Finally, the NESHAP for Combustion Sources [11] was promulgated in 2002 and controlled HAP emissions from the combustion sources at pulp mills.

The Clean Air Act (CAA) requires EPA to review, and if appropriate, revise the NSPS and the NESHAP every eight years. With the changing market and the potential to reduce emissions when reviewing the pulp and paper sector regulations, it would be beneficial to have a model that can analyze the impacts of potentially more stringent emission limits and new emission standards for pollutants not previously regulated on individual mills as well as the U.S. and the global market. This model would assist EPA in determining the impacts of revised federal regulations, as well as obtaining an estimate of how mills might comply with the new standards (e.g., reduced production, process changes, add-on controls, fuel switching, equipment upgrades, or mill closure).

### Industrial Sector Integrated Solutions Model

The ISIS model was initially used to analyze multi-pollutant regulatory options for the cement industry [3]. Subsequently, EPA’s Office of Research and Development (ORD) and Office of Air Quality Planning and Standards (OAQPS) expanded the ISIS modeling framework to include the pulp and paper sector. The ISIS pulp and paper framework identifies pulp mills and paper mills as two independent units. As a result, the ISIS input database is divided into two major sections: pulp data and paper data. This renders the flexibility to independently optimize the pulp mill from the paper mill operation at an integrated facility, and vice versa. All mills with available data that have operated for at least one year since 2000 are represented in the database. Contrary to the cement model, the pulp and paper model does not project new mills or options for the addition of new production capacity by region, because no new integrated mills have been built since 1990, and the current trend is to reopen closed mills. Each facility modeled in ISIS is characterized by its location, pulping process, equipment utilized, annual product capacities, cost components, and retirement information when available. This information is discussed further in the following sections.

The current version of the ISIS pulp and paper model focuses on the combustion sources located at pulp and papermaking facilities; these sources are recovery furnaces, lime kilns, and boilers. Recovery furnaces are used to recover valuable pulping chemicals for re-use in the process and to generate process steam. Similarly, boilers are used to generate steam and electricity for the facility and its processes. Lime kilns are used to convert the recovered pulping chemicals into fresh pulping chemicals. It is necessary to relate control costs and potential emission reductions of these units to production of a given commodity. For pulp and paper, this is not a direct relationship because these sources do not produce the final product, but rather perform a supporting function of energy production (boilers) and chemical recovery (recovery furnaces and lime kilns). Recovery furnaces and lime kilns in particular are associated with the pulping process rather than papermaking. This article will discuss the data collection and database development needed to link emissions from boilers, recovery furnaces, and lime kilns to pulp and paper production.

## Data Collection

The inputs to the ISIS model for the pulp and paper sector can be broadly categorized into the following main components: final product data, mill-level data, cost data, demand modeling data, import modeling data, emissions and controls data, and regulatory options. These data sets were compiled into a database and used to populate the ISIS model input sheet.

Data to assist in characterizing individual facilities and the collective industry were purchased from Resource Information Systems, Inc. (RISI) for facilities with available data. RISI works with industry and trade associations to collect information for their databases and is considered to be the leading information provider for this industrial sector [12]. RISI data are commonly used in U.S. and global market modeling for the pulp and paper sector; for example, the U.S. Department of Agriculture (USDA) and the Department of Energy recently utilized RISI data to model U.S. biomass supply curves for biofuel production [13]. In another example, the Canadian Forest Service used RISI data to model supply and demand of the forest sector in British Columbia in response to bioenergy utilization (pellet production and energy generation) [14]. This data is publically available for purchase on the RISI website, but is not shared here due to purchase restrictions. Data on pulp and paper production, cost, facility characterization, product composition, import, and export were processed using these datasets. The data collections purchased from RISI and a description of the collections are as follows:

- North American Graphic Paper Capacity Report 2011—this report summarized the current, planned, and future capacity for the North American printing and writing paper market [15].
- North American Graphic Paper Historical Data—this report contained 17 years (1994 to 2010) of historical data (annual basis), including production, consumption, imports, exports, capacity, prices, and costs for the graphic papers, recovered paper, and pulpwood markets [16].
- World Recovered Paper Annual Historical Data—this report contained 17 years (1994 to 2010) of historical data (annual basis) for supply, demand, and price for recovered paper [17].
- North American Paper Packaging Capacity Report 2011—this report summarized the current, planned, and future capacity for the North American paper packaging market [18].
- North American Paper Packaging Annual Historical Data—this report contained 17 years (1994 to 2010) of historical data (annual basis), including production, consumption, imports, exports, capacity, prices, and costs for corrugated box, containerboard, boxboard, packaging and industrial papers, and recovered paper and pulpwood [19].
- World Market Pulp Capacity Report 2011—this report contained current, historical, and future capacity for the world paper grade market pulp industry [20].
- World Pulp Annual Historical Data—this report contained 17 years (1994 to 2010) of historical data (annual basis) for the world market pulp industry [21].
- World Tissue Capacity Report 2011—this report contained current, historical, and future capacity of the world tissue market [22].
- United States Mill Asset Database—this database provided process flow diagrams for U.S. pulp and paper facilities [23].

The 2011 Lockwood-Post, a directory of the North, Central, and South American pulp and paper mills, was also purchased from RISI [24]. This directory was a compilation of survey information obtained annually from many of the mills and companies listed, supplemented with data from other sources. The data in the directory included facility locations, process types, product types, and production capacities. Only facilities operating in 2011 and those which closed in 2010 were included in the directory (i.e., facilities that where idle or closed prior to 2010 were not included and neither were those in an idle state in 2011, but expected to reopen in 2012).

Emissions and controls data were obtained from an information collection request (ICR) survey [25] sent to the pulp and paper industry by the EPA in 2011. This survey collected information for use in the following current and future regulatory reviews: the review of the NESHAP for Pulp and Papermaking Sources, the NESHAP for Chemical Combustion Sources, and the review of the NSPS for Kraft Pulp Mills. Only major source facilities (i.e., those that emit, or have the potential to emit, 10 tons per year or more of any hazardous air pollutant (HAP) or 25 tons per year or more of any combination of HAPs) in operation during base-year 2009 were required to complete the survey. According to the survey, the most common HAPs emitted from pulp and paper facilities were methanol, acid gases, acetaldehyde, and formaldehyde. Data for smaller sources known as area source facilities (not a major source per the definition in the CAA section 112) were not collected. The area source facilities were typically stand-alone paper mills that utilized market pulp or recycled paper as their raw material. Data collected in the ICR survey are available through the www.regulations.gov docketing system, docket numbers EPA-HQ-OAR-2007–0544 and EPA-HQ-OAR-2012–0640.

Historical mill information was obtained from the “Mills Online” database [26] maintained by the Center for Paper Business and Industry Studies (CPBIS) at the Georgia Institute of Technology. This database is publically available and provides historical mill data for all facilities which have operated in the U.S. since 1970. The data included mill location, number of products produced, and whether or not a facility was an integrated facility. The website also tracks company announcements such as capacity expansions, closures, and acquisitions.

Supplemental data for facilities were obtained from trade organization websites, such as, the mill curtailments and closures spreadsheet maintained by the Pulp & Paperworkers' Resource Council [27], and corporate websites. These data were used to determine if facilities were closed and to determine product composition (e.g., recycled material content). Data for a total of 514 facilities were collected using these various sources.

## Data Processing

### Finished Product Data

The ISIS pulp and paper modeling efforts were focused on representing the entire population of U.S. integrated and non-integrated pulp and paper mills and their products. Subcategories of paper products provided by RISI were grouped into eight major categories, primarily classified on the basis of product end use. Similarly, there were two major categories of pulp products (softwood and hardwood pulp). Table 1 lists the major categories and subcategories of the pulp and paper products utilized in the model.

**Table 1:** ISIS product categories for the U.S. pulp and paper market.

Paper products		
Major Category	Subcategories	
Containerboard (CNT)	Bleached Kraftliner	Recycled Medium
	Unbleached Kraftliner	Recycled Liner-board
	White-top Kraftliner	White-top Recycled Liner
	Semichemical Medium	
Boxboard and Other Board (BXT)	Bleached Boxboard	Coated Cartonboard
	Unbleached Boxboard	Uncoated Cartonboard
	Folding Cartonboard	Gypsum Wallboard Facings
	Liquid Packaging board	Tube, Can, Core, and Drum
	Food Service (cup and plate)	Multi-ply/Multi furnish
	Folding Boxboard	Boxboard
	Other Unbleached Boxboard	White-lined Chipboard
	Recycled Boxboard	Liquid Packaging board
	Other Recycled board	Bleached Kraft board
Packaging and Industrial Paper (PIP)	Kraft Wrapping Paper	Unbleached Packaging Paper
	Unbleached Kraft Paper	Bleached Packaging Paper
	Bleached Kraft Paper	Specialty and Industrial Paper
Corrugating Medium (COR)	Semichemical Medium	Recycled Medium
Newsprint (NEW)		
Tissue (TIS)		
Coated Printing and Writing Paper (CPW)	Coated Freesheet	Coated Mechanical
	Coated Bristol	Coated Groundwood
Uncoated Printing and Writing Paper (UPW)	Uncoated Freesheet	Fiber Paper
	Uncoated Bristol	Uncoated Bristol and Cotton
	Cotton Fiber Papers	
**Pulp Products**		
Hardwood Pulp (HWPLP)	Bleached Hardwood Kraft	Mechanical Hardwood
	Unbleached Hardwood Kraft	Mechanical Softwood
Softwood Pulp (SWPLP)	Bleached Softwood Kraft	Unbleached Softwood Kraft

### Mill Level Process Characterization

The number of mills in the ISIS database and their locations were determined using the North American Graphic Paper Capacity Report, the North American Paper Packaging Capacity Report, and the World Tissue Capacity Report purchased from RISI. The mill types (integrated vs. non-integrated) were determined using the ICR and CPBIS’s “Mills Online” database and the Mill Asset Database. For mills that are currently closed, a retirement date was determined using the Pulp & Paper Resource Council’s website and the capacity report (i.e., facilities with “0” capacity were assigned the first year of “0” capacity as their retirement year). Facilities in the ISIS database were identified based on a plant type (“PR” for paper mill or “PL” for pulp mill) and an ISIS ID number. These values were concatenated to form mill identification numbers and were then used to identify the facilities in the model.

The United States Mill Asset Database, a collection of process flow diagrams for 232 facilities, was used to extract process and production information. The data extracted included pulp production, product composition (i.e., grade recipes), facility type (i.e., whether or not the facility could purchase market pulp and/or recycled pulp), number of boilers and recovery furnaces, fuels utilized, steam and electricity generation, and electricity utilization. These data were extrapolated to the remaining 282 facilities that did not have flow diagrams available based on products produced at the facility, mill type, and region. Production process information for facilities without process flow diagrams (e.g., the ability to process recycled fiber) was determined using company websites.

The final product-specific capacity for each facility was determined using the maximum production value for the 10-year period for which data was purchased. It was assumed that a facility was operating at 85-percent capacity in the year with the highest production (based on utilization rates provided in the ICR). This value represents the maximum amount of each product a facility can make without making a process change or upgrading equipment. Capacity for a facility was reported as “0” for years which production values were “0,” as these periods represent periods after a mill was closed, stopped producing a particular product, or before obtaining equipment to produce the specific product. An example of the calculations are shown in Table 2.

**Table 2:** Production capacity example.

Values (1000 ton/year)	2000	2001	2002	2003	2004	2005	2006	2007	2008	2009	2010	Max
**Production**	5.5	7.5	6.0	8.5	10.5	7.5	5.5	5.0	2.5	0	0	10.5
**Capacity**	12.4	12.4	12.4	12.4	12.4	12.4	12.4	12.4	12.4	0	0	12.4

For integrated facilities, pulp production and pulp capacity were calculated using the process flow diagrams for those with available data. For pulp production, pre-digester and post-digester pulp values were extracted and used to calculate pulping yield. For example, a pre-digester value of 2400 bone dry short tons per day (BDST/D) of softwood chips and a post-digester value of 1170 BDST/D of softwood pulp indicate a pulping yield of 48.75-percent. This pulping yield was then used in conjunction with the values in the product summary table on the process flow diagrams for the amount of wood used for each product to determine the amount of pulp needed for each product. This was repeated for all products produced at a mill, and then summed to obtain the final pulp production value. Final pulp production values were averaged based on the major product category produced, and tons of pulp needed per ton of product values were assigned to similar facilities where process flow diagrams were unavailable. Pulp mill capacity was determined using the maximum production year and a utilization factor of 85-percent, the same method used to calculate final product capacity in Table 2.

Product composition was determined using the product summary table on the process flow diagram for facilities where flow diagrams were available. These values represented the amount of softwood pulp, hardwood pulp, additives, and recycled pulp in the final product. In the case of integrated facilities, the pulping yield, as calculated for the pulp mill production and capacity values, was also used to determine product composition. For non-integrated facilities, values for pulps and recycled papers from the flow diagram summary table were used to calculate product composition. All recycled paper values (e.g., deinked pulp, pulp substitutes, old corrugated containers, old newsprint, and mixed papers) were summed to create one “recycled pulp” value for each product. Market pulp values (e.g., northern bleached softwood kraft pulp and northern bleached hardwood kraft pulp) were maintained as separate values to determine softwood and hardwood percentages. Average product compositions were determined based on final product category and mill classification (e.g., non-integrated vs. integrated, recycle mill vs. virgin mill) and assigned to facilities without flow diagrams.

It was confirmed that the paper grade recipes matched the classification of the facility. For example, a facility producing boxboard from 100 percent recycled material was confirmed to be represented by a “1” in the “facility can purchase recycled pulp” column. It was also confirmed, for example, that a facility producing tissue from 50-percent hardwood pulp and 50-percent softwood pulp could produce the pulp (indicated by a “Y” in the “integrated facility” column) or could purchase the pulp (indicated by a “1” in the “can purchase market pulp” column) for use in the final product. In cases where these conditions were not met, product recipes were adjusted accordingly; that is, if a product composition was assumed for a facility and that facility could not produce or purchase virgin pulp, but could purchase recycled pulp, the product composition was changed to 100-percent recycled. Some facilities produce both products from recycled pulp and products from virgin pulp. In these cases, all products were compared to the mill classification to confirm that the facility was capable of making all of the products based on their characterization. It was also confirmed that all pulp mills had corresponding pulp production data. For example, an integrated facility producing hardwood pulp and making containerboard from hardwood and softwood pulp was confirmed to be represented by “Y” in the “integrated facility” column and a “1” in the “facility can purchase pulp” column. For the integrated mills, it was confirmed that hardwood pulp production and/or softwood pulp production values were available. If all years were reported as zero for both pulp types, the pulp mill classification was removed and the facility was classified as a stand-alone paper mill. An example of these classifications can be seen in Table 3, for all three types of facilities (i.e., stand-alone paper [PR540], stand-alone pulp [PL812], and integrated [PR291/PL291]).

**Table 3:** Mill classification example.

ISIS Facility ID	Integrated?	Paper Capacity Available	Market Pulp Capacity Available	Can Produce Market Pulp?	Can Purchase Market Pulp?	Can Purchase Recycled Pulp?
PL291	Y	NA	Y	1	NA	NA
PR291	Y	Y	NA	NA	0	0
PR540	N	Y	NA	NA	1	1
PL812	N	NA	Y	1	NA	NA

Pulp mill type was determined for the 163 pulp mills in the database. This information dictated the type of equipment utilized at the facility, as well as the type and quantity of emissions. Pulp mills were classified as mechanical or chemical based on the process flow diagrams, ICR information, and company websites. Chemical pulp mills were further classified as sulfate (kraft pulping), the predominant chemical pulping process utilized in the U.S., or other pulping process.

Emission unit data were determined using the ICR, the Boiler Maximum Achievable Control Technology (MACT) database [28], and the process flow diagrams. For integrated facilities, boilers were assigned to paper mills, while lime kilns and recovery furnaces were assigned to pulp mills. The average number of boilers, lime kilns, and recovery furnaces (three, one, and two, respectively) was determined for integrated facilities with data and were assigned to those without data. The average number of boilers (two) for stand-alone paper mills was assigned to facilities without flow diagrams. Lime kilns and recovery furnaces were not assigned to facilities using chemical pulping methods other than sulfate (kraft), unless ICR data showed that the facility had one or both.

Existing controls for the assigned equipment were determined based on the ICR and the Boiler MACT database for those with data available. Boiler controls were assigned based on fuel type and products produced at similar facilities with data. Lime kiln controls were assumed to be scrubbers for all kilns without data, and recovery furnace controls were assumed to be dry-bottom electrostatic precipitators, both based on the representative control for the majority of emission units with data.

Lime mud production and black liquor production were calculated using information collected in the ICR. Values in the ICR for lime mud production were based on a ton of calcium oxide produced per day basis, and a recovery rate of 90-percent was assumed to calculate the amount of lime mud burned per day. This value was then combined with the previously calculated daily pulp production for 2009, resulting in a tons of lime mud produced per ton of pulp produced (TLM/TP) value. An average value of 0.270 TLM/TP was assigned to mills without ICR data (this value was the average of all available data). Black liquor values in the ICR were based on million pounds of solids per day. This value was combined with the previously calculated daily pulp production for 2009, resulting in tons of black liquor solids produced per ton of pulp produced (TBLS/TP). An average value of 1.49 TBLS/TP value was assigned to mills without ICR data (this value was the average of all available data).

Landfill data were provided in the summary table of the process flow diagrams in the form of ton of waste per ton of final product. Waste materials generated at the facility and disposed of in a landfill for this database are typically sludges generated from paper recycling and wastewater treatment. For facilities without a process flow diagram, a landfill value was assigned based on mills with data of similar mill type and final product type. This data is needed to estimate the cost of disposing of waste materials, and could potentially be used to estimate emissions from the landfill.

Electricity data were extracted from the boiler section of the process flow diagrams for the available facilities. In this section, the number of power boilers was indicated for each plant, as well as the amount of electricity generated, used, purchased, and sold. These values were combined with the daily production values to determine the electricity values (i.e., generated, used, purchased, and sold) per ton of finished product. Facilities with electricity values were averaged based on all of the products produced at each facility, as well as the facility type and then applied to similar facilities producing similar products without process flow diagrams.

Boiler data was extracted from the boiler section of the flow diagrams for available facilities. The number of power boilers and the fuel used for each facility was extracted. Fuel intensities, amounts of each fuel used per ton of production, and electricity usage were also extracted from the flow diagram summary table. Some boilers had input fuels that were not assigned to product intensities. These fuel intensities were not populated; however, they were included as fuels available for use by the facility. Fuel intensities were averaged per product and assigned to facilities without flow diagrams. Controls and boiler fuel types were assigned based on the most frequently used for each product in the database. Boiler fuel data as well as the number of boilers were also extracted from the Boiler MACT database and compared to the flow diagram results. These additional data assisted in populating values for facilities without flow diagrams. Facility fuel availability was assigned to each facility based on fuel intensities from the flow diagram summary table and boiler input fuels (flow diagram and Boiler MACT). These values were assigned to facilities without process flow diagrams based on final products produced and facility type.

### Cost Data

Cost data were obtained from RISI or calculated for six primary cost functions in the model. The primary cost functions were raw material cost, maintenance and repair cost, labor cost, fuel cost, electricity cost, and solid waste disposal cost. The development of these functions is discussed in this section.

#### Raw Material Cost

Hardwood and softwood logs and chips serve as raw materials for integrated facilities and non-integrated pulp mills, whereas market pulp and recycled paper serve as the raw materials for non-integrated paper mills. The ISIS pulp and paper model identifies paper mills and pulp mills as two separate units, regardless of whether or not the facility is integrated. Raw material cost for pulp mills included the cost of raw wood, pulping chemicals and wastewater treatment, whereas, paper mill raw material cost represented the cost of papermaking chemicals and purchased fiber (i.e., recycled fiber and market pulp). Total raw material cost ($/short ton of finished product/year) for each major product was calculated by adding the cost of individual raw material cost components mentioned above. Individual raw material costs were obtained from the RISI database for a period of 11 years (2000 to 2010). Finally, the average raw material cost for each major product was calculated by summing the weighted average (based on the capacity in respective year) of each year’s total raw material cost.

#### Maintenance and Repair Cost

Repair and periodic maintenance are required for facility upkeep. Maintenance and repair costs ($/short ton of finished product/year) for each major product category were obtained from the RISI database for a period of 11 years (2000 to 2010). RISI reported the maintenance costs for integrated mills only. Reported maintenance costs were equally divided between independent pulp mills and stand-alone paper mills to conform to the ISIS pulp and paper design framework. Average maintenance and repair cost for each major product was calculated for individual mill by adding the weighted average (based on the capacity in respective year) of each year’s maintenance and repair cost.

#### Labor Cost

Total labor costs ($/short ton of finished product/year) were obtained by adding operating labor cost and mill-salaried labor costs. Labor costs ($/short ton of finished product/year) were calculated based on the data reported by RISI Inc. for a period of 11 years (2000 to 2010), but the RISI database represented the labor costs for integrated mills only. Therefore, 48 percent of the total labor costs were assigned to the paper mills, and 52 percent was assigned to the pulp mills [29]. Average labor cost for each major product was calculated for individual mills by adding the weighted average (based on the capacity in respective year) of each year’s labor cost.

#### Fuel Cost

Coal, oil, natural gas, black liquor, biomass, hog-fuel sludge, bark, and tire derived fuel (TDF) are the primary fuels used in the pulp and paper industry. Coal, oil, natural gas, biomass, hog-fuel, bark, sludge, TDF are largely consumed in the boiler whereas, black liquor is primarily used in the recovery furnace. The ISIS database gives a detailed description about the types of fuels used in each pulp and paper mill in the United States. The fuel database of ISIS was constructed based on the information collected from RISI Inc. and the ICR database. Fuel cost ($/million British Thermal Units) in each state for each fuel type has been collected from the U.S. Energy Information Administration website [30].

#### Electricity Cost

Electricity is consumed primarily by the auxiliary equipment and paper machine(s). Integrated facilities often produce more electric power than required and sell extra electricity to the power grid. The ISIS database reported the amount of electricity sold, produced and/or purchased with respect to each mill in the United States. Electricity cost (cents/kilowatt-hour) in each state was collected from the U.S. Energy Information Administration [31].

#### Solid Waste Disposal Cost

Facilities must dispose of production process waste materials and many do so in private landfills. The amount of waste generated per ton of finished product was calculated as discussed previously and a value of $50 per waste ton was assigned as the disposal cost [32].

### Demand Modeling Data

#### Demand Centers

The pulp and paper industry is regional in nature. In ISIS, each facility modeled is located in one of three regional markets, as defined in the report published by USDA in 1994 [33]. These regional markets are north, south, and west. The ISIS pulp and paper model allows for all modeled facilities to supply demand in any region, subject to transportation costs as discussed later. It is important to note that the purchased RISI data divided the market into two regional markets (north and south) as opposed to the three regional markets. However, it was decided that three regional markets mentioned by in the USDA report [33] would be a better representation of the U.S. market because transportation is treated similarly. Data for the mills that belonged to the northwest and southwest regions in the RISI database were assigned to the mills located in the west region of the ISIS database.

#### Transportation and Interregional Trade

**Fig 2:**
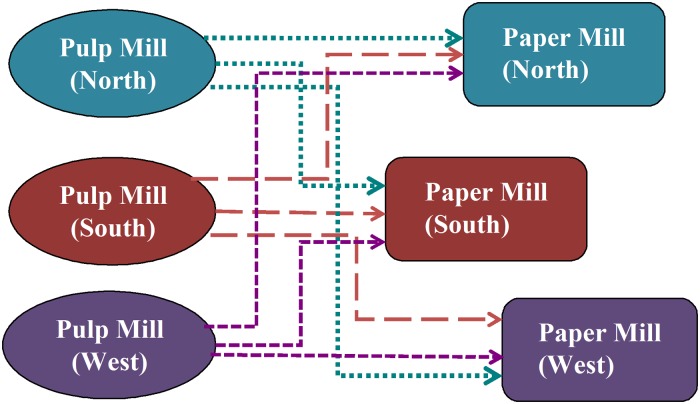
Domestic transport of pulp from pulp mills to paper mills.

**Fig 3:**
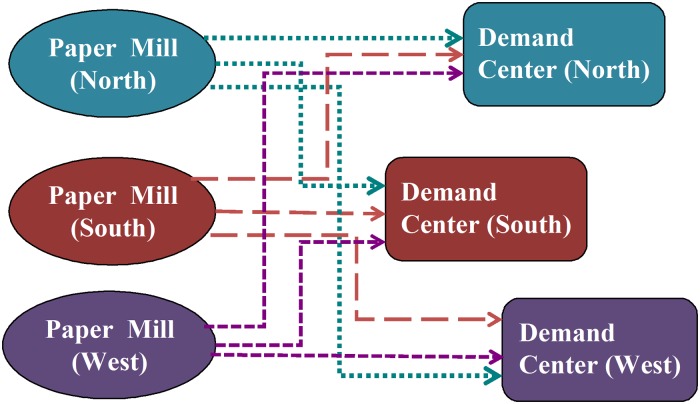
Domestic transport of paper products from paper mills to demand centers.

In the ISIS pulp and paper model, a domestic transportation matrix was used to describe the costs for transporting pulp from pulp mills to paper mills, and from paper mills to demand centers (the U.S. market for paper products). Figs. 2 and 3 illustrate the ISIS network of domestic transport of pulp and paper in three regions within the United States. Fig. 2 describes the domestic trade of softwood and hardwood pulps from pulp mills to paper mills located in three designated ISIS regions (north, south, and west). Similarly, Fig. 3 shows the transport of the eight major paper products from paper mills to demand centers or U.S. market.

Figs. 2 and 3 demonstrate that there is no constraint in the supply route. In other words, the ISIS database allows all regional pulp and paper mills to supply all regional paper mills or demand centers, respectively. This signifies the flexibility of the ISIS model to select the most effective transportation route (based on cost) to obtain the optimal solution.

Transportation costs were calculated by adapting the dollar value published in the USDA “Recycling and Long-Range Timber Outlook” report [33]. However, transportation costs in the USDA report were reported as 1986 dollar value. Inflation was taken into account and the real 1986 dollar values were adjusted by the consumer price index published by the Bureau of Labor Statistics [34] to obtain real 2010 dollar values to represent the 2010 inter-regional transportation in the ISIS database.

### International Trade Data

**Fig 4:**
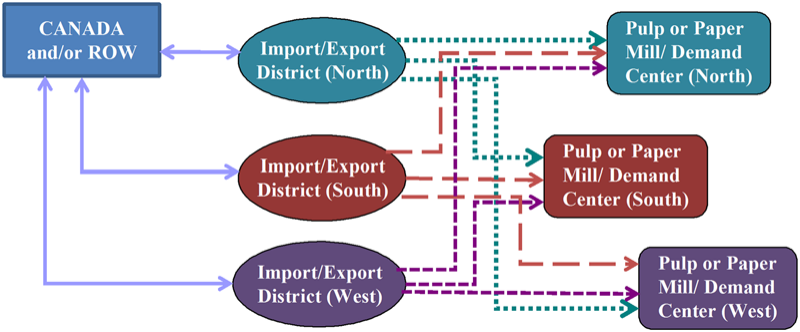
Trade network of pulp and paper products from Canada and/or Rest of the World to pulp/paper mills and demand centers respectively.

The U.S. pulp and paper markets trade pulp and paper products with a number of countries; however, the U.S. trades significant quantities of pulp and paper products with Canada. Considering Canada’s role in trade with the United States, international trade in pulp and paper products was simplified to represent trade between Canada, the rest of the world (ROW), and the U.S. These traded products arrive and leave from three geographical domestic regions: north, south and west, as illustrated in Fig. 4. In Fig. 4, pulp is imported from the Canada and/or ROW market to pulp mills via import districts and then distributed to paper mills. Pulp is exported to Canada and/or ROW through export districts. Fig. 4 also demonstrates that paper is imported from Canada and/or ROW through import districts to regional demand centers. Paper is exported from the paper mills to Canada and/or ROW through the export districts. The average quantities of traded pulp and paper products were determined based on the data reported by RISI Inc., for a period of 11 years (2000 to 2010). Transportation costs between regions and mills and/or demand centers were also reported in the database.

### Emissions and Controls Data

The design of the ISIS pulp and paper model can accommodate any number of pollutants of interest. In the model, each boiler, recovery furnace and lime kiln can be characterized by its nitrogen oxides (NO_X_), sulfur dioxide (SO_2_), hydrochloric acid (HCl), mercury (Hg), PM, volatile organic compound (VOC), total hydrocarbon (THC), total HAP, and carbon dioxide (CO_2_) emissions. The first phase of the model includes the following pollutants: NOx, SO_2_, and PM. The second phase will consider HCl, VOC, THC, Hg, and total HAP.

In the ISIS pulp and paper model, pollution control technologies were related to boiler, recovery furnace, and lime kiln heat inputs and/or furnace gas flow rate. The NO_X_ emissions from the pulp and paper industry result primarily from the boilers and the recovery furnace, and reduction methods included in the model were low NOx burners (LNB), ultra-low NOx burners, over fire air (OFA), flue gas recirculation (FGR), selective non-catalytic reduction (SNCR), selective catalytic reduction (SCR), and low temperature oxidation [35].

Sulfur dioxide emissions from the recovery furnace are the product of sulfur in the smelt, and SO_2_ emissions from power boilers, especially those firing coal or residual fuel oil, are directly related to the sulfur content of the fuel. Wet and dry scrubbing technologies may be applied in the model to control SO_2_ emissions.

The ISIS pulp and paper model database contains information on abatement approaches for NO_X_, SO_2_, and PM emissions. The four categories of abatement approaches included are: add-on controls, combustion modifications and upgrades, fuel switching, and mitigation boiler/furnace technologies. Add-on control abatement approaches include flue gas desulfurization, spray dryer absorber, and in-duct dry sorbent injection methods for SO_2_ control, and SCR and SNCR methods for NO_X_ control in power boilers. Flue gas desulfurization abatement method in recovery furnace is used for SO_2_, and SCR and SNCR abatement methods in recovery furnace are used for NO_X_. Combustion modification used in power boiler are LNB, FGR, OFA, oxygen trim and water injection, and upgrade combustion controls for NO_X_. For each emission abatement approach, where possible, information on the following parameters were developed [35] and included in the database: capital cost, fixed operating cost, variable operating cost, emission reduction performance for all of the pollutants, impacts on fuel and/or raw material use, impact on electricity consumption, byproduct generation and cost, and impact on water use.

To estimate capital recovery factors for capital costs associated with control technologies, economic life values and interest rate were considered. Economic life for each of these measures can be taken to be the average of the technical life and the payback period. Again, an interest rate of seven percent can be used for capital recovery in the absence of more specific information. End users can select different values for economic life and interest rate as appropriate.

### Policy and Economic Parameters

The ISIS model can be used to analyze the impact of new or modified air regulations on individual facilities and on the industrial sector. Traditional policy scenarios in the model can include unit-specific emission limits based on production rates or concentrations. For example, a revision to the PM emission limit for recovery furnaces could be imposed in the model to determine several parameters such as the number of units that could not meet the new standard, how units could comply (e.g., process changes, add-on controls, reduced production), impacts to the general population such as product price increases, and impacts to the global market. The model can also analyze cap-and trade policy scenarios, if needed. Currently in the ISIS database, the following economic parameters are defined: discount rate, future escalation rates of various energy, material, transportation, and labor inputs, demand elasticities for each major category of products, and ten years of demand projections (tons/yr) for each major category of products.

The demand projections for each major product are key components in the model. This is because the demand for products drive production levels at mills in the model, and changes in costs resulting from regulatory scenarios adjust demand levels via a price mechanism. Estimating future demand of pulp and paper products required a two-step statistical modeling process. First, supply and demand systems were estimated using the historical trends in the product data compiled for the ISIS model. These models permitted an examination of the sensitivity (or “elasticity”) of product demand to various potential explanatory variables. These models served as the bases for the second step of the future demand modeling in which the explanatory variables where forecast into the future and, along with the parameter estimated for the demand equation, a baseline forecast of each product type was discerned. In the model, as a result, product demand varies from the baseline forecast as consumers potentially face different market prices for products as mills adjust to the simulated regulatory impacts.

In estimating supply and demand equations, the convention in economic statistics that supply and demand systems are simultaneously determined was followed. In other words, the supply and demand equations for each given product must share the same market clearing price, therefore, a change in quantity supplied simultaneously affects the market price and, as a result, causes a change in demand. This process is non-recursive, meaning that feedback travels in both directions, and presents statistical challenges in that the supply and demand equations are correlated through their error terms. The simultaneity of the supply and demand equations also introduces an additional “identification’ challenge, which is the inability to identify individual supply and demand curves, as the observed data used in the models only depict market clearing prices under the finite set of observed conditions, not the range of possible conditions which may be of interest from a regulatory or policy perspective.

To resolve these challenges, a two-stage least squares (TSLS) approach using instrumental variables was utilized. The TSLS approach helps to avoid the biased estimation of the supply and demand model parameters by isolating the exogenous behavior of price in the pair of equations for each pulp and paper product. The statistical strategy additionally leverages an instrumental variable (IV), which is an exogenous variable that is correlated with price but not the error terms of either model. The IV or “shifter” affects demand conditions without affecting cost conditions or could affect cost conditions without affecting demand conditions. These “shifters” are used to trace out or identify the supply or demand curve.

After estimating the supply and demand models, these models were used to develop baseline forecasts for pulp and paper products. To do so, however, required forecasts of the explanatory variables in the demand models. For some variables, the data sources from which the explanatory variables were drawn already had forecasts, such as those available from macroeconomic models, such as Gross Domestic Product. For other variables, a technique from time series analysis called autoregressive integrated moving average (ARIMA) modeling was utilized. The ARIMA models rely upon trends in the variable of interest alone to obtain forecasts, as opposed to attempting to explain influences upon the variable and estimating important elasticities as done in the supply and demand modeling.

## Model Input Data Summary

Finished product data was collected for 514 facilities and 37 product subcategories. Many facilities produced more than one subcategory of each grade of paper, and, as a result, a total of 908 sets of 10-year data were available for the 8 major paper products and the 2 major pulp products. The uncoated printing and writing (UPW) category had the most information with 211 data sets at 146 facilities, and contained subcategories such as uncoated mechanical, uncoated freesheet, uncoated groundwood, uncoated bristol, and cotton fiber papers. The remaining categories can be found in Table 4, which summarizes the number of datasets available for analysis and the number of facilities producing each product.

**Table 4:** Summary of production by major product category.

Major Category	Number of Data Sets	Number of Facilities
Uncoated Printing and Writing Paper	211	146
Boxboard and Other Board	170	135
Packaging and Industrial Paper	143	34
Tissue	86	86
Containerboard	77	73
Corrugating Medium	70	66
Coated Printing and Writing Paper	68	58
Newsprint	24	24
Softwood Pulp	39	39
Hardwood Pulp	20	20
**TOTAL**	**908**	**N/A**

Production for the major paper products (as categorized in the ISIS model) for 2000 and 2010 are shown in Table 5. All of the products except for tissue (TIS) experienced a decline during this time period. Newsprint (NEW) experienced the largest decline with production dropping from 7.5 million tons to 3.6 million tons, a decrease of 52 percent. Overall industry production declined from 105 million tons to 90 million tons, a reduction of 14.5 percent.

**Table 5:** Summary of production by major product category.

Major Category	2000 Production (million ton/year)	2010 Production (million ton/year)	Change (%)
Tissue	7.4*	7.6	2.7
Containerboard	26.4	25.6	-3.0
Corrugating Medium	11.0	10.4	-5.5
Packaging and Industrial Paper	6.2	5.6	-9.7
Boxboard and Other Board	17.4	14.8	-14.9
Coated Printing and Writing Paper	11.1	8.8	-20.7
Uncoated Printing and Writing Paper	18.2	13.5	-25.8
Newsprint	7.5	3.6	-52.0
**TOTAL**	**105.0**	**90.0**	**-14.5**

*This value is for 2005, the first year of available data for TIS in the RISI report

**Fig 5:**
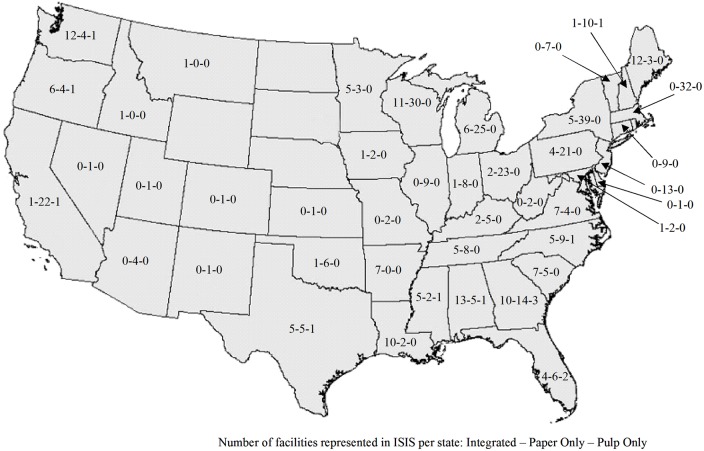
Map of pulp and paper facilities representing in ISIS model.

The total number of facilities represented in ISIS was 514, which consisted of 151 integrated facilities, 12 stand-alone pulp mills, and 351 stand-alone paper mills. Individually, there were 163 pulp mills and 502 paper mills represented in the model. These facilities were located in 43 states, divided into three regions. There were 63 facilities located in the west region, 160 facilities in the south, and 291 facilities in the north. Of the 163 pulp mills, 145 were classified as chemical pulp mills and 18 were classified as mechanical pulp mills. According to the populated retirement dates, 346 of the facilities were operating in 2012. Fig. 5 shows the number of facilities by type in each state that is represented in the database.

Facilities were classified as to whether or not they could purchase recycled fiber and produce or purchase market pulp (i.e., pulp produced at a facility and sold in the pulp market instead of being utilized on site). Of the 163 pulp mills, 49 mills were able to produce market pulp. Many paper mills, 322, were able to purchase recycled fiber, and 193 were able to purchase market pulp.

The electricity data showed that 513 facilities consume electricity. The remaining facility utilized only steam according to the RISI data. Many facilities, 391, were able to produce electricity, and 19 of those produced a surplus of electricity and sold the excess to the grid. A total of 490 facilities purchased electricity, and the average electricity consumed for all facilities was 899 kWh/ton product (this includes purchased and produced electricity).

Fuels such as coal and natural gas may be available to a facility even if it is not currently in use. Fuel availability was determined for all facilities so that fuel switching could be utilized as an emission reduction strategy. Table 6 shows the number of facilities with the option to use each type of fuel assessed.

**Table 6:** Fuel availability summary.

Fuel	Number of Facilities
Natural Gas	488
Oil	335
Coal	210
Hog	171
Free hog	142
Sludge	34
Tire-derived fuels (TDF)	19
Pet-coke	14

Boilers were assigned to paper mills and stand-alone pulp mills in the model to prevent duplicate counting. All of the facilities in the database except for two had at least one boiler and a total of 1,196 were included in the model. Of the 163 pulp mills, 128 were assigned at least one recovery furnace and 124 were assigned at least one lime kiln. A total of 217 recovery furnaces and 162 lime kilns were included in the model.

**Fig 6:**
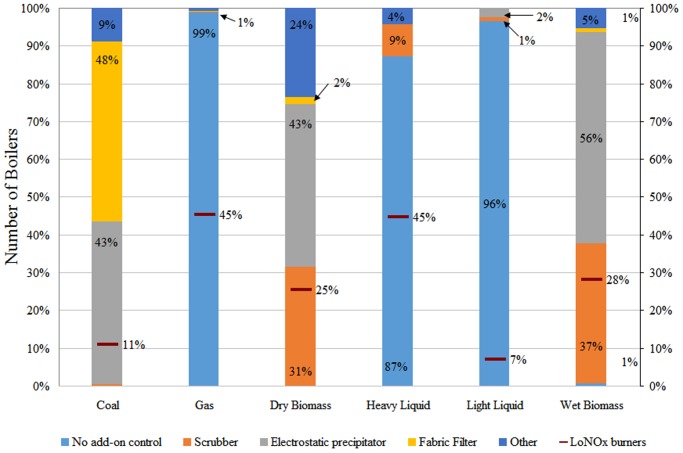
Air pollution controls for boilers utilizing different fuel types.

Existing controls were assigned to the boilers, recovery furnaces, and lime kilns based on ICR data and assumptions previously discussed. For recovery furnaces, dry-bottom electrostatic precipitators (ESPs) were the most commonly utilized air pollution control device (155 units). Wet-bottom ESPs were second most common, utilized on 40 recovery furnaces. Dry-bottom ESPs with wet PM return systems were utilized on 16 units, while combinations of ESPs and scrubbers were used on six units. For lime kilns, a scrubber was the most commonly used air pollution control device (121 units), followed by an ESP at 27 units. Fourteen additional units utilized combinations of cyclones, ESPs, and scrubbers. Boiler controls were specific to fuel type and are shown in Fig. 6.

**Fig 7:**
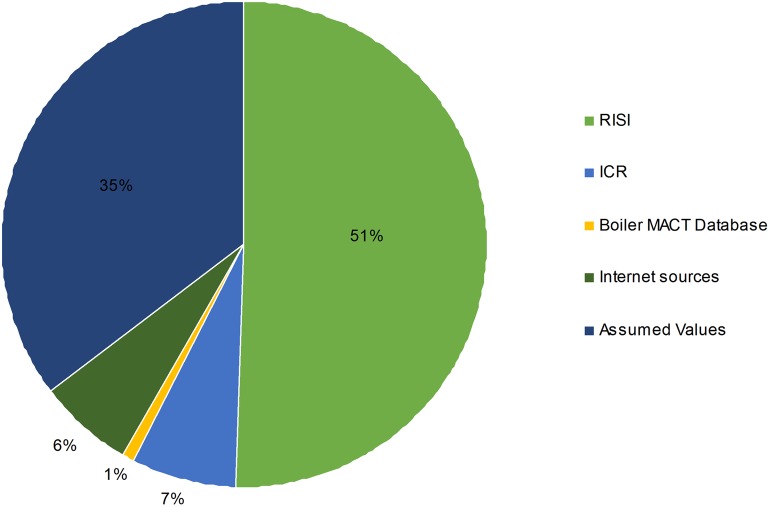
Percentages of available data from each source utilized in developing the database.

In summary, approximately 51-percent of the data in the database were based on the data purchased from RISI. These data elements included product composition, facility characterization and location, historical pulp and paper production values, fuel types, and pulping yields. Approximately 35-percent of the data was based on assumptions made from the RISI and ICR data, and the remaining 14-percent were data from the ICR, the boiler MACT database, and other internet sources. These percentages are shown in Fig. 7.

## Data Utilization in the Model

**Fig 8:**
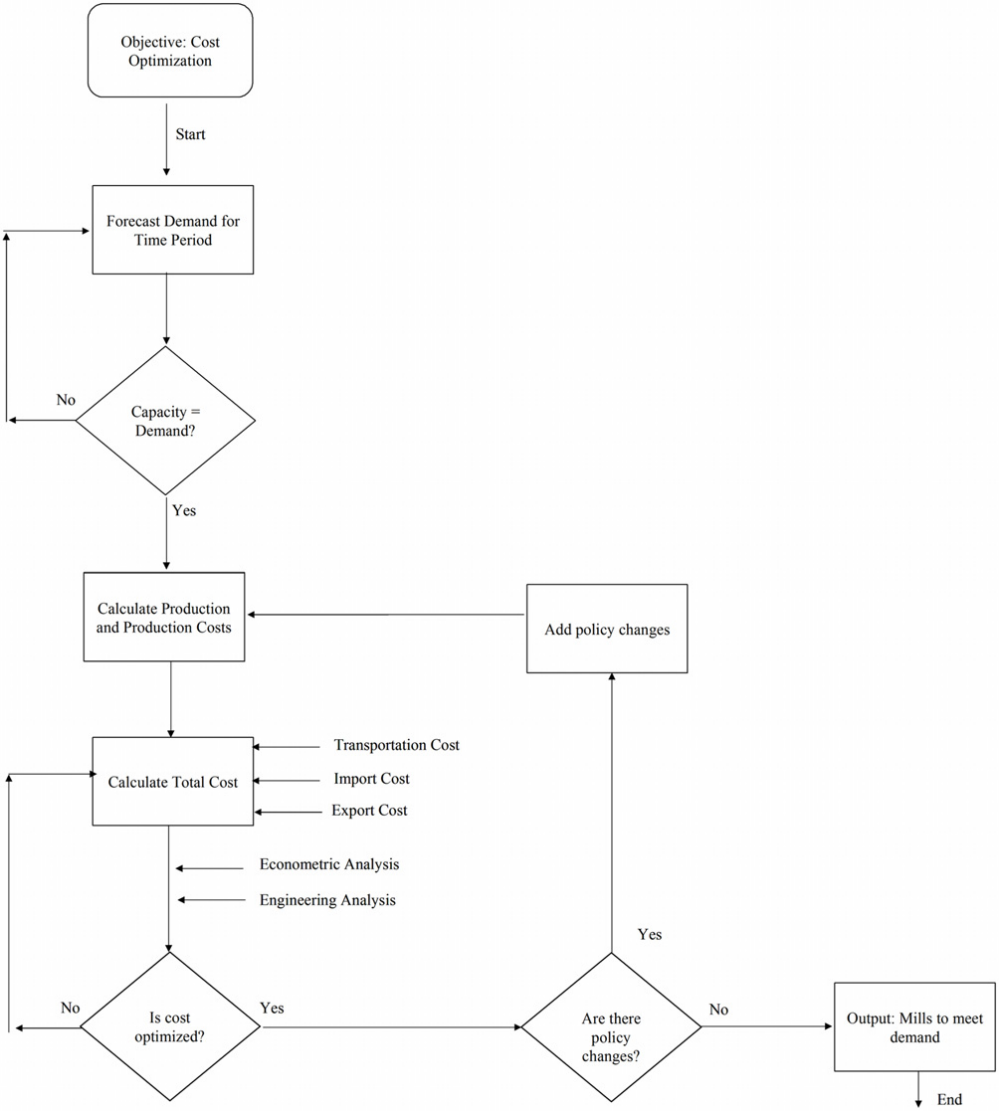
Algorithm used in ISIS model describing the cost optimization process.

**Fig 9:**
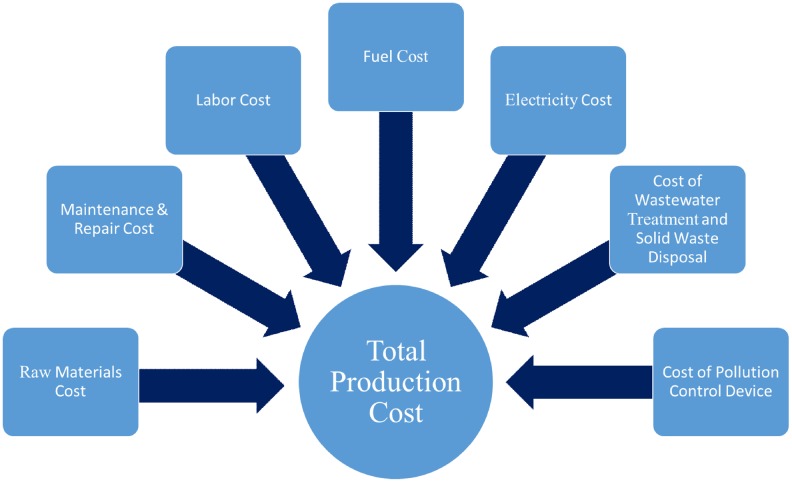
Different cost components used in ISIS model for performing production cost Calculation.

The extensive amount of data discussed in this article are used to characterize the individual facilities and the U.S. and global markets. The objective of the model, to optimize cost, is achieved using the algorithm in Fig. 8. First, the model forecasts demand for a given time period using demand projection curves. Then, the model confirms that the industry has capacity to meet the projected demand. Once this condition is met, the model calculates production from each facility and their production costs. This step utilizes the raw material, labor, maintenance, and the energy costs for each plant to determine production cost, (Fig. 9) as well as the pulping yield, product composition, and facility characterization data to determine how facilities meet demand. In the case of mills producing pulp, calculated pulp production numbers are used in conjunction with the tons of lime mud and black liquor solids produced to calculate emissions (to be used when a policy change occurs). Total cost is then calculated by adding transportation costs, and/or import or export costs when appropriate. The model then determines if the cost is optimized, and if it is not, it repeats the process until the most cost effective facilities meet the projected demand. Once cost is optimized, policy changes can be used as a constraint and the model will repeat the production and cost calculations until optimized. The final output is which mills are used to meet the demand based on their cost performance.

## Model Utilization

The ISIS model can be a useful tool for regulatory development by simulating the potential impacts of alternative environmental policy on the pulp and paper sector. With dynamic market conditions and the potential to improve efficiency and effectiveness of existing emission standards when reviewing the sector regulations, it is beneficial to have a model that can analyze potential impacts on individual mills as well as the potential effects on domestic and international markets.

As part of the regulatory development process, the EPA performs an economic assessment that generally contains following analyses: engineering cost, market, economic welfare, and small entity analyses. The complexity of these analyses generally follows the significance of the regulatory action and may also include qualitative and quantitative analyses of potential benefits from the action. The engineering cost analysis estimates the total annualized engineering costs incurred by the affected facilities annually to meet new or modified requirements. In the ISIS model, regulatory parameters are entered into the model exogenously and the cost incurred by each facility to meet a new standard is estimated endogenously by the model as behavior of producers and consumers adjust to a new equilibrium. In ISIS, the market analysis follows from the decision-making in the model as market impacts (prices and quantities) can be compared with and without the simulated regulation. Welfare analysis can follow from the market analysis in that consumer and producer surplus can be estimated, which are informative indicators of the magnitude and distribution of economic impacts across firms or consumers. As required by the Regulatory Flexibility Act, the EPA also performs an analysis of the relative burden of regulatory actions on impacts on small businesses, small governments, and other small organizations. This analysis is typically initiated by comparing estimated annualized engineering compliance costs at the firm-level, which can be summed across plants under common ownership in ISIS, to firm sales.

## Conclusions

The ISIS database serves as a comprehensive information source for the ISIS pulp and paper model. The objective of this extensive database is to provide an accurate representation of the U.S. pulp and paper industry so that the EPA can evaluate and optimize the best mitigation option to reduce emissions. The database contained detailed information on processes utilized, product characterization, cost, and demand data inputs that enable the model to run comparable engineering and economic analyses. Furthermore, the database was constructed to enable the model to support the decision making for regulatory development and impacts assessment.

## Future Work

A future goal for ISIS database is to provide more extensive information on emission characterization and intensity, energy use, demand parameters, and characterization of control technology. This will be done by testing assumptions, and filling data gaps based on industry feedback for emissions, controls, consumptions, and applicable costs. The EPA would maintain the database by updating the mill specific data to keep the ISIS pulp and paper model entirely representative of ongoing scenario of the U.S. pulp and paper industrial sector. Eventually, additional processes and emission points will be added to the model for use in other future regulatory reviews.

## Disclaimer

Although this work was reviewed by the EPA and approved for publication, it may not necessarily reflect official agency policy. Reference herein to any specific processes or services by trade name, trademark, manufacturer, or otherwise does not necessarily constitute or imply its endorsement, recommendation, or favoring by the U.S. government. The views and opinions of authors expressed herein do not necessarily state or reflect those of the U.S. government and shall not be used for advertising or process endorsement purposes.
